# Differences in health behaviours between immigrant and non-immigrant groups: a protocol for a systematic review

**DOI:** 10.1186/2046-4053-3-61

**Published:** 2014-06-10

**Authors:** Suresh Joshi, Santosh Jatrana, Yin Paradies, Naomi Priest

**Affiliations:** 1 Alfred Deakin Research Institute (ADRI), Faculty of Arts and Education, Deakin University, Geelong Waterfront Campus, Locked Bag 2000, 3220 Geelong, VIC, Australia; 2 Centre for Citizenship and Globalization (CCG), Faculty of Arts and Education, Deakin University, 221 Burwood Highway, 3125 Burwood, VIC, Australia

**Keywords:** Immigrant, Non-immigrant, Foreign-born, Nativity, Health behaviour, Tobacco smoking, Physical activity, Alcohol drinking, Systematic review

## Abstract

**Background:**

Health behaviours are important determinants of health and adoption of unhealthy behaviour is considered as one of the mechanisms through which immigrants’ health changes over time in the host country. The change in health behaviours over time can contribute either to improving or worsening the overall health status of immigrants. Despite being the important mediators for the change in overall health status and chronic health conditions, no previous review (either general or systematic) has examined differences in key health behaviours simultaneously between immigrants and non-immigrants. This study aims to provide a systematic overview of the current global literature on differences in key health behaviours (that is, tobacco smoking, physical activity and alcohol drinking) between immigrant and non-immigrant groups.

**Methods/Design:**

Empirical studies in English language reporting quantitative data simultaneously on both immigrant and non-immigrant groups will be considered for this systematic review. Electronic scientific searches will be conducted on seven databases to identify relevant studies of interests: MEDLINE, CINAHL, PsycINFO, EMBASE, Global Health, SocINDEX and ProQuest. In addition, Google/Google Scholar will be used to find the relevant studies and personal contact with experts will also be undertaken. Titles, abstracts and keywords of studies identified in the search strategies will be screened for inclusion criteria. The authors will select the studies following the PRISMA guidelines. The quality of included studies will be appraised using the Critical Appraisal Skills Programme (CASP) checklists. A descriptive summary statistics of included studies will describe the study designs, socio-demographic characteristics, and the exposure (immigrant and non-immigrant groups) and outcome (key health behaviours) measures. *P*-values and confidence intervals (CIs) for the associations between exposure and key health behaviours will also be reported.

**Discussion:**

This systematic review will facilitate a better understanding of differences in key health behaviours between immigrant and non-immigrant counterparts. It will provide a rigorous and reliable research base for future research and advance information on key health behaviours for a range of immigrant groups compared to non-immigrants in the high-migrant-receiving countries.

**Systematic review registration:**

This systematic review protocol has been registered with PROSPERO (registration number: CRD42014008688).

## Background

Immigrants’ health behaviour is known to be influenced by the new environment and the culture in the host countries [1]. The empirical research on immigrants health behaviours compared to non-immigrants originates predominately from the United States, Canada and the United Kingdom. The existing observational research suggests a lower prevalence of smoking, physical activity and risky alcohol drinking among immigrants of Hispanic, African and Asian origins to the United States [2-8], lower prevalence of smoking and risky alcohol drinking among various immigrant groups in Canada [9,10] and lower prevalence of smoking and risky alcohol drinking among Asian and African immigrants as compared to UK-born counterparts [11].

Acculturation, the process of adopting attitudes, values, customs, beliefs and behaviours of another culture, may lead to change in health behaviour of immigrants over time [2,12]. The effect of acculturation for immigrants can be associated either positively or negatively for the change in health behaviours. For example, Abraido-Lanza et al. [2] suggest that Latinos relative to non-Latino whites were less likely to smoke and drink alcohol. However, as the duration of residence (one of the measure of acculturation) increases in the United States, Latinos were significantly more likely to adapt unhealthy behaviours such as tobacco smoking and binge drinking [2]. On the other hand, smoking is more prevalent among foreign-born (FB) Asian men relative to US-born Asian men [7]. Over time these rates are thought to converge to host country levels, thus effectively lowering the overall prevalence of smoking among Asian immigrants [13]. The change in these health behaviours overtime thus can contribute either to improve [4,14] or worsen [9,15,16] the overall health status of immigrants.

Health behaviours are significant risk factors for the overall health status and chronic health conditions such as cardiovascular diseases (CVD), diabetes, obesity and cancers [16-18]. There are limited reviews of the literature on immigrants’ health, with existing reviews focusing on specific health problems among particular immigrant groups in the host countries [19-21]. Despite the role of health behaviours in changing the health (either improving or worsening) of the population including immigrants, no previous review (either general or systematic) has examined differences in key health behaviours between immigrant and non-immigrant groups. Without this comparison, the different immigrant trajectories in health behaviour cannot be attributed to immigrant status.

### Aims and objectives

This review article will provide the first international systematic review of empirical studies over the two decades of the relative degree to which key health behaviours (that is, tobacco smoking, physical activity and alcohol drinking) differs between immigrants (and subgroups) and the non-immigrants, and how that changes with duration of residence. Specifically, this systematic review aims to: (1) examine the key characteristics of quantitative studies comparing health behaviours of immigrants with non-immigrants including where and when studies have been conducted, age and gender of study populations, nativity status, study designs, sample sizes and data sources used; (2) assess the differences in health behaviour prevalence between immigrants and non-immigrants across countries; and (3) determine whether the duration of residence in the host country is associated with the change in health behaviours among immigrants. The ultimate goal of this comprehensive systematic review is to identify the potential gaps in the existing literature on immigrants’ key health behaviours and to generate important recommendations for future research directions.

## Methods

### Design

The design of this research will be a systematic review. This systematic review will adopt and follow the reporting guidelines and criteria set in Preferred Reporting Items for Systematic Review (PRISMA) statement which is known to ensure the highest standard in systematic reviews [22].

### Inclusion and exclusion criteria for considering studies

#### ***Study design or types of studies***

In this systematic review we will include peer-reviewed journal articles (includes published articles and articles in press) and dissertations/theses. Empirical research studies using quantitative study design and research methods will be included. Such studies will include cross-sectional; cohort (prospective and retrospective); case–control; longitudinal studies; experimental or interventional study designs (randomized controlled trials (RCTs), cluster-randomized trials (CRTs), quasi-randomized trials); and non-randomised controlled studies (non-randomized controlled trials, controlled before-after studies (CBAs) and interrupted time series studies (ITSs) and historically controlled studies). This systematic review will only extract the quantitative data if any study that meets our inclusion criteria has a mixed-methods design (that is, both quantitative as well as qualitative data). Research articles based on commentaries of the literature (opinion articles), editorials, literature reviews, systematic reviews, meta-analysis, blogs, newsletters, organisation documents and government reports, book/book chapters, and conference abstracts or proceedings will be excluded.

#### ***Populations of interest and exposure measures***

This study will review studies reporting an empirical analysis that involves a comparison of immigrants with non-immigrant control groups. According to the definition of International Organisation for Migration (IOM), immigrant can be defined as foreign-born person who has moved to another country for the purpose of settlement [23]. The above definition of immigrant may include economic migrants, temporary foreign workers, foreign students, documented and undocumented migrants, refugees and asylum seekers [23]. Reported nativity (for example, whether a person is foreign-born or native-born) will be considered as a measure of exposure for this systematic review. Therefore, based on the reported nativity of the study participants in the included studies, immigrant and non-immigrant groups will be determined. However, studies reporting data either only on immigrant or on non-immigrant groups (that is, not a comparative study) will be excluded. Additionally, studies focusing on internal migrants will also be excluded for this systematic review. Only adults aged 18 years and older will be included as study participants in this systematic review. However, there will be not any restrictions for the gender and the geographical location (immigrants’ country of origin and destination) of the study participants.

#### ***Outcome measures***

Key health behaviours (that is, tobacco smoking, physical activity and alcohol drinking) will be the outcome measures in this systematic review. Outcomes focusing on knowledge of and attitude towards tobacco smoking, physical activity, drinking and other health behaviours will not be considered. Other health behaviours such as health screening, sexual behaviours, diet/nutrition and seat belt use will be excluded for this systematic review.

### Search strategy

The following databases will be used to search for relevant publications: MEDLINE, EMBASE, CINAHL, PsycINFO, Global Health, SocINDEX and ProQuest (for dissertations/theses). Additionally, Google/Google Scholar will also be used to find the relevant studies available in full text. Manual hand-searching of reference lists from studies identified as relevant will be conducted to locate further articles and dissertations of interest. One of the authors will consult the experts in this field including the first author of the key articles to identify any other relevant studies [24]. All searches will be limited to studies published in the English language between 1994 and 2014. Additional file 1 provides the full description of the search terms and search strategy for MEDLINE database which will be adapted and modified where necessary for other databases.

To ensure maximum sensitivity and specificity when retrieving articles, controlled terminologies such as subject headings, keywords, treasure terms and text-words will be searched using a systematic process. For example, while searching the MEDLINE database, the Medical Subject Headings (MeSH) will be used to find the relevant literature. All search terms will be ‘truncated’ and ‘exploded’ to ensure all associated terms are included in the database search. Duplicate articles will be removed. Database search will be performed with a frequent consultation with a qualified medical research librarian.

### Identification and selection of studies

Initially, two authors will independently screen relevant citations (title, abstract and keywords). The first author will subsequently screen the full text of articles and dissertations meeting inclusion criteria at title/abstract screening level. The other three authors will independently screen one-third each to ensure a full double-review. For any disagreements during the screening process with regards to inclusion/exclusion of studies will be resolved by consensus and/or by discussion between all the authors. Rationale for excluding studies will be recorded and reported as part of the screening process [24]. The authors will select the studies following the evidence based checklists developed by the PRISMA statement [22]. PRISMA statement are developed to provide clear, practical and systematic guidelines in researching and writing systematic reviews. The PRISMA statement consists of an evidence-based checklist of 27 items [22]. Additionally, based on PRISMA guidelines [22], a flow diagram will also be developed to show the process of study selection through different phases. Full text studies meeting the inclusion criteria will be either directly or manually imported and stored in Endnote X7 (Thompson Reuters, CA, USA).

### Data extraction and management

An excel spreadsheet will be used for extracting data and to collect details from the publications that meet the inclusion criteria. The first author will initially extract the information from all publications with data extraction for 10% of included studies conducted independently by the second, third and fourth authors. Disputes regarding data extraction will be resolved by the discussion between all the authors. The reviewers will attempt to contact the authors by e-mail if required to obtain essential missing data from the studies that meet our inclusion criteria. The PROGRESS-Plus framework [25] will be used to record the methodological variables, socio-demographic characteristics, and the exposure and outcome measures obtained from the selected studies. Table 1 shows the list of variables for data extraction.

**Table 1:** Variables to be extracted based on the PROGRESS-Plus framework

**Methodological variables**	**Socio-demographic characteristics**	**Exposure measures**	**Outcome measures**
Authors	Age	Nativity status	Key health behaviours:
Year of publication	Race/ethnicity		
Type of publication	Gender		1. Prevalence of tobacco smoking
Study year	Education		
			2. Prevalence of physical activity
Study design (including sampling procedure)	Income		
Sampling characteristics	Employment status		
1. Sample size (including immigrant groups size)			
	Language use		
2. Study location (country of destination for immigrants)			3. Prevalence of alcohol drinking
	Immigrant (for example, Asian, African, European) and non-immigrant groups		
Time-point (for measurement of outcomes when reported more than once)	Duration of residence for immigrants (if available)		

### Methodological quality assessment

The quality of each eligible study will be assessed using the validated critical appraisal tools adapted from the Critical Appraisal Skills Programme (CASP) questions for observational studies developed by Health Evidence Bulletin Wales [26]. The key domains of the CASP checklist used to determine the quality of the studies are clarity of aims, appropriate and rigours methods and analysis, as well as the risk of bias, and the significance (value) of research. Quality for all included studies will be assessed by the first author and for a 10% of included studies checked for completeness and accuracy independently by the second, third and fourth authors. Differences in the quality assessment will be resolved by discussion between all the authors.

### Data synthesis and reporting the findings

The data synthesis will begin with a descriptive summary statistics of the study characteristics extracted from the included studies. A summary table on risk of bias for the included studies will also be provided. Key themes related to health behaviours (tobacco smoking, physical activity and alcohol drinking) will be reported and discussed separately. Where possible, dichotomous (or binary) outcomes will be expressed as relative risks (RR), odds ratio (OR) or risk difference (RD). For continuous outcomes, we will report means or mean differences between the groups. For longitudinal studies, the effect size to be reported will be the estimates of the change in health behaviours over time. If available, *P*-values and confidence intervals (CIs) for the associations between exposure and key health behaviours will also be reported for the included studies.

Meta-analysis will only be considered when the included studies are sufficiently homogeneous in terms of study design, participants, interventions and outcomes to provide a meaningful summary measures. The I^2^ statistic will be used to assess the level of heterogeneity present in the included studies, and meta-analysis will not be performed if I^2^ is > 50% [24]. In the presence of moderate heterogeneity (I^2^ = < 50%) between the included studies we will pool the results using a random-effects model, with standardised mean differences (SMDs) for continuous outcomes and odd ratios (OR) or risk ratios (RR) for binary outcomes [24]. If meta-analysis is not possible, the data will be synthesised narratively based on a framework for narrative synthesis [27]. A funnel plot will be used to visually assess the presence of publication bias in the included studies [24]. The strengths and limitations of the included studies will be taken into account qualitatively and reported in the discussion part of this systematic review. Study recommendation for policy and potential further research possibilities will also be reported.

## Discussion

### Strengths and limitations of the review

This is the first systematic review particularly aimed at determining the current global literature on differences in key health behaviours between immigrant and non-immigrant groups. Strengths of this systematic review include a clear inclusion and exclusion criteria, and an advanced and transparent systematic approach for searching, screening, appraising and extracting the current literature as well as summarising data using standardised tools at all stages. However, this study will also involve judgements made by review authors, which can lead to reporting bias. There are some other limitations for this systematic review. First, the search will be limited to studies in the English language between 1994 and 2014, thus we cannot report findings from important studies written in other languages and prior to 1994. Second, by restricting the participants to adults (aged 18 years and above), we will be unable to report the prevalence of smoking, risky drinking and physical activity behaviours of children and adolescents. Third, we are only focusing on empirical research studies based on quantitative measures, which will limit to report the findings from studies using qualitative measures (that is, focus group discussions and key informants interviews). Finally, we are also excluding current literature on other health behaviours such as health screening, sexual behaviours, diet and nutrition, and seat belt use which are also significant health risk behaviours for both immigrant and non-immigrant groups.

### Implications for research and policy

This systematic review will provide a rigorous and reliable research base for future research and advance information on key health behaviours for a range of immigrant groups compared to non-immigrants in the high-migrant-receiving countries. It can also help the healthcare policy makers to prepare and implement health behaviour change interventions such as smoking cessation, encouraging regular physical activities for both immigrant and non-immigrant population.

### Dissemination

The goal of our dissemination is to produce key information for policy makers and other stakeholders to develop effective evidence-based healthcare interventions, education and health promotion programmes to both the immigrants and non-immigrants of high-migrant-receiving countries. The findings from this anticipated review will be disseminated for scientific peer-reviewed publications as well as conference presentations and proceedings. The review authors will also disseminate the findings to the health researchers and academic institutions through national and international workshops/seminars.

## Abbreviations

CASP: Critical Appraisal Skills Programme; FB: Foreign-born; IOM: International Organisation for Migration; PRISMA: Preferred Reporting Items for Systematic Review.

## Competing interests

The authors declare that they have no competing interests.

## Authors’ contributions

SJ conceived and designed the study. SJ drafted the manuscript and led the development of the search strategy. SJ (second author) contributed to the overall study design, preparation of the study protocol, developing the search strategy, and preparation of draft. YP and NP provided important inputs regarding the methodology and revised the protocol for important intellectual content. All authors critically reviewed the manuscript and approved the final version submitted for publication. All authors read and approved the final manuscript.

## Supplementary Material

Additional file 1 Search strategy for MEDLINE database. Click here for file
