# Supplementary material for: Differences in health behaviours between immigrant and non-immigrant groups: a protocol for a systematic review
Source: Syst Rev. 2014 Jun 10;3:61. doi: 10.1186/2046-4053-3-61 (PMC4062644; doi:10.1186/2046-4053-3-61)
Supplement: Additional file 1 — Search strategy for MEDLINE database. [file 2046-4053-3-61-S1.pdf]

Additional file 1: Search strategy for MEDLINE database (will be adapted for other databases as needed)

EBSCOhost interface will be used to perform MEDLINE database search. Screen search will be performed using the advanced search option.

Search terms:

1. Transients and Migrants **(MeSH)**

2. Emigrants and Immigrants **(MeSH)**

3. Emigration and Immigration **(MeSH)**

4. migrant* OR emigrant* OR immigrant* OR migrat* OR emigrat* OR immigrat* OR “migrant worker*” OR foreigner* OR “foreign-born*” OR refugee* OR “asylum seeker*”

5. ethnic* OR “ethnic group*” OR “minority group*” OR nativity OR nationalit* OR “country of birth” OR newcomer* OR “new-comer*” OR “cross-cultur* comparison*” OR “culturally and linguistically diverse” OR CALD

6. 1 OR 2 OR 3 OR 4 OR 5

7. Tobacco Use **(MeSH)**

8. Smoking **(MeSH)**

9. smok* OR “tobacco smok*” OR “tobacco use” OR “tobacco consumption*” OR “cigarette smok*”

10. 7 OR 8 OR 9

11. Exercise **(MeSH)**

12. Running **(MeSH)**

13. Jogging **(MeSH)**

14. Swimming **(MeSH)**

15. Walking **(MeSH)**

16. “physical* activ*” OR “physical* inactiv*” OR exercise* OR “physical exercise” OR “sedentary behav*” OR sedentar* OR “leisure-time physical activit*” OR sport*

17. 11 OR 12 OR 13 OR 14 OR 15 OR 16

18. Alcohol Drinking **(MeSH)**

19. Alcoholism **(MeSH)**

20. Alcoholic Beverages **(MeSH)**

21. Binge Drinking **(MeSH)**

22. “alcohol consumption” OR “alcohol use” OR “alcohol drink*” OR “alcohol abuse” OR “ethanol drink*” OR “binge drink*” OR “heavy drink*” OR drink*

23. 18 OR 19 OR 20 OR 21 OR 22

24. “health behav*” OR “health risk behav*” OR lifestyle* OR “life-style” OR “life style*”

25. 10 OR 17 OR 23 OR 24

26. Epidemiologic Research Design **(MeSH)**

27. Epidemiologic Studies **(MeSH)**

28. “cross-section*” OR “cross section*” OR “cross-sectional stud*” OR “case-control” OR “case control” OR cohort* OR “cohort* analysis” OR longit* OR “longitudinal stud*” OR “longitudinal survey*” OR “follow-up” OR “follow up” OR “follow-up stud*” OR prospective OR retrospective OR quantitative OR survey* OR trial* OR intervention* OR “randomised control* trial*” OR “randomized control* trial*” OR “before and after” OR “interrupted time series” OR questionnaire* OR registr* OR evaluat* OR audit*

29. 26 OR 27 OR 28

30. 6 AND 25 AND 29

31**.** Limit 30 to (Full Text; Date of Publication= “1994 - 2014”; Academic Journals; English Language; and Human)
